# High prevalence of *Coxiella burnetii* infection in humans and livestock in Assiut, Egypt: A serological and molecular survey

**DOI:** 10.14202/vetworld.2020.2578-2586

**Published:** 2020-12-05

**Authors:** Hypy Abbass, Salah Abdel Kareem Selim, Mona M. Sobhy, Mohamed A. El-Mokhtar, Mahmoud Elhariri, Hanan H. Abd-Elhafeez

**Affiliations:** 1 Department of Microbiology, Microbiologist at South Egypt Cancer Institute of Assiut University. Egypt; 2 Department of Microbiology, Faculty of Veterinary Medicine, Cairo University, Egypt; 3 Department of Reproductive Diseases, Animal Reproduction Research Institute, Animal Research Centre, Giza, Egypt; 4 Department of Medical Microbiology and Immunology, Faculty of Medicine, Assiut University, Assiut 71515, Egypt; 5 Department of Microbiology, Faculty of Veterinary Medicine, Cairo University, Giza, Egypt; 6 Department of Anatomy, Embryology and Histology, Faculty of Veterinary Medicine, Assiut University, Egypt

**Keywords:** apparently healthy farm animals and humans, *Coxiella burnetii*, enzyme-linked immunosorbent assay, hepatitis C and B, immunofluorescence assay, Q fever, real-time quantitative polymerase chain reaction

## Abstract

**Background and Aim:**

Q fever is considered a neglected zoonotic disease and is caused by *Coxiella burnetii*. Very little information is available on *C. burnetii* infections in cattle, sheep, and goat populations in Egypt. The aim of this study was to identify the seroprevalence of *C. burnetii* in humans and livestock and to test for the presence of *C. burnetii* DNA in sera from seropositive animals and humans.

**Materials and Methods:**

Blood samples were collected from 160 apparently healthy farm animals and 120 patients from three hospitals of the Assiut Governorate throughout 2017/2018. These populations were tested for antibodies against *C. burnetii* phase II antigen by immunofluorescence assay [IFA] and enzyme-linked immunosorbent assay (ELISA). Seropositive samples were subjected to real-time quantitative polymerase chain reaction (RT-qPCR).

**Results:**

The results of the IFA revealed *C. burnetii* seroprevalence rates of 45.3%, 56.0%, 45.7%, and 53.3% in cattle, sheep, goats, and humans, respectively. In humans, the seroprevalence rates were 52.1%, 30.4%, 37.5%, 74.1%, and 62.5% in patients with fever of unknown origin, influenza, kidney dialysis, hepatitis C virus, and hepatitis B virus, respectively. Likewise, by ELISA, the seroprevalence in bovine was 50.7%; sheep, 60.0%; goats, 51.4%; and humans, 55.0% (54.3%, 30.4%, 37.5%, 77.8%, and 62.5% in patients with fever of unknown origin, influenza, kidney dialysis, hepatitis C virus, and hepatitis B virus, respectively). RT-qPCR targeting the repetitive element IS1111 confirmed the presence of *C. burnetii* DNA.

**Conclusion:**

These results proved that apparently healthy cattle, sheep, and goats may be very important reservoirs of *C. burnetii* infection. In light of these data, the effect of Q fever on the replication of hepatitis virus remains unclear. Although hepatitis is one of the main aspects of acute Q fever, the influence of hepatitis on Q fever remains to be investigated. Q fever is not a reportable disease in Egypt, and clinical cases may rarely be recognized by the health-care system. Additional information on the epidemiology of *C. burnetii* in Egypt is warranted, including other associated problems such as the distribution of infections, pathologic hallmarks, and molecular typing.

## Introduction

Fever may be a symptom of infection with *Coxiella burnetii*, a small obligate intracellular Gram-negative pathogen spreading worldwide, except in New Zealand [1-4]. Sheep, goats, and bovines are considered the main reservoir of human infection [5,6]. In animal hosts, abortions, and stillbirths are among the foremost pathological manifestations of chronic Q fever [7-9]. In sheep and goat flocks with reproductive disorders, animals may shed the bacterium in vaginal mucus, feces, and milk [10]. Sheep, goats, and bovid are the main subclinical carriers, but they can shed bacteria in various types of secretions and excretory products. In a recent study, goats were shown to eliminate *C. burnetii* mostly through their milk, whereas sheep eliminated the bacterium through their vaginal mucus or feces [11]. *C. burnetii* was the cause of abortion waves at 28 dairy goat farms and a couple of dairy sheep farms in the Netherlands [12,13]. Infection may persist for many years and may be lifelong. Humans are usually infected through airborne transmission from animal reservoirs, particularly from domestic ruminants [14].

People living in or next to farms are at increased risk of acquiring infection due to potential contact with infected animals, and people working in laboratories are also at risk because of contact with potentially infected organs and tissues [15].

Infection is usually transmitted by inhalation of desiccated aerosol particles and through contact with infected animals, animal tissue, or other animal products, such as wool [14]. Because *C. burnetii* can be secreted in the milk, the consumption of contaminated food such as raw milk and dairy farm products represents a route of infection for humans [14]. Clinically, acute Q fever in humans may present with flu-like symptoms usually followed by pneumonia, whereas chronic infection may involve endocarditis and death [16].

*C. burnetii* undergoes phase variation during antigenic transition from wild-type phase I to virulent phase II throughout serial passages in embryonated eggs or in cell cultures [14]. Serology assays can detect antibodies in phase I and phase II of *C. burnetii* infection. Phase II antibodies typically prevail throughout infection, whereas chronic infection is characterized primarily by a phase I antibody response [17]. Indirect immunofluorescent assay (IFA) can be used in the serodiagnosis of Q fever [18-21] and may be applicable not only in diagnosing Q fever and its therapeutic follow-up but also in screening sera in massive numbers [15,22]. So far, seroprevalence data on the incidence of current infection in humans or animals are limited. The methods used for the identification of *C. burnetii* strains include nested polymerase chain reaction (PCR) [23], real-time quantitative PCR(RT-qPCR) [24], touch-down PCR [25], and trans-PCR targeting IS1111, the repetitive transposon-like region of *C. burnetii* [26]. These tools are very helpful for epidemiological investigations, especially for linking sources of infection, for higher understanding of epidemiological risk factors, and to a lesser extent, for evaluating control measures.

Little information is available regarding *C*. *burnetii* infections in bovid, sheep, and goats in Egypt. Therefore, the aim of this study was to identify the seroprevalence of *C. burnetii* by IFA and to detect the presence of *C. burnetii* DNA in samples from seropositive animals, which could be a source of *C. burnetii* transmission.

## Materials and Methods

### Ethical approval and informed consent

The National Ethics Committee of Assiut University and Cairo University and the Veterinary authorities in Assiut and Cairo Provinces approved this study. Informed consent was obtained from human participants.

### Sampling

Blood samples were collected from apparently healthy animals, including 75 bovids, 50 sheep, 35 goats, and 120 humans (from three hospitals). The blood samples were collected from the brachial vein of humans and the jugular vein of the animals. The samples were collected under aseptic conditions from randomly selected farms in different localities in the Assiut Governorate, Egypt, during 2016/2017. Serum samples were transferred into sterile vacuum tubes and stored at −20°C until processed [14].

### Indirect IFA for the detection of anti-*C. burnetii* antibodies

For the IFA, we used a commercially available kit (COXIELLA BURNETII I+II IFA IgG/IgM/IgA, Vircell). Serum samples were tested for IgG and IgM anti-*C. burnetii* phase II antibodies using slides coated with *C. burnetii* phase II antigen. The IFA was carried out as recommended by the manufacturer. Serial two-fold dilutions of serum were prepared in phosphate-buffered saline with a goat antihuman IgG-fluorescein isothiocyanate (FITC) immunoconjugate, IgM-FITC immunoconjugate, anti-*C. burnetii* antibody-positive rabbit serum, and anti-*C. burnetii* antibody-negative rabbit serum. The slides were examined by fluorescence microscopy under ultraviolet light using ×400. The samples emitting a green fluorescent color at a titer ≥1:64 were considered positive [14,27].

### Enzyme-linked immunosorbent assay (ELISA) for the detection of anti-*C. burnetii* antibodies

ELISA kits were used for indirect multi-species ELISA-based detection of anti-*C. burnetii* antibodies in serum and milk samples from multiple host species (ID Screen® Q Fever Indirect Multi-species, product code FQS-MS-2P [lot number: C32]; ID-Vet, Gabrels, France).

The presence of anti-*C. burnetii* IgM antibodies are highly suggestive of recent or active *C. burnetii* infection. Meanwhile, the presence of anti-*C. burnetii* IgG antibodies indicate the previous infection [28].

### RT-qPCR

#### Extraction of DNA

For whole-blood samples, DNA purification was performed using the QIAamp® DNA Mini Kit (Qiagen, Hilden, Germany, Cat. no. 51304) according to the manufacturer’s instructions [29].

### RT-qPCR assay optimization and validation

#### Primer design

The primers were selected based on published sequences of the transposon-like repetitive region of the *C. burnetii* genome (IS1111 gene). The IS1pri_f (5′-CGCAGCACGTCAAACCG-3′) and Is1Pri_r (5′-TATCTTTAACAGCGCTTGAACGTC-3′) primers were used for qPCR together with the Tqpro_Is1 probe (FAM-5′-ATGTCAAAAGTAACAAGAATGATCGTAAC-3′-TAMRA) [29]. The oligos were obtained from Metabion, Germany.

### PCR optimization and validation

Conventional PCR was used to improve the sensitivity of the current target RT-qPCR assay for *C. burnetii* DNA. Conventional PCR was performed in a final volume of 50 μL of reaction mixture containing 25 μL of DreamTaq Green PCR Master Mix (Thermo Fisher Scientific, Waltham, MA, USA) [30], 0.8 μL of 25 pmol of each IS1pri_f and IS1pri_r, 5 μL of extracted DNA, and water sufficient to make up the reaction mixture volume. The following conditions were applied: Initial hot start at 95°C for 5 min, followed by 40 cycles of denaturation at 95°C (30 s), annealing at 60°C (30 s), and elongation at 72°C (30 s). The cycle was finalized with elongation at 72°C for 5 min. PCR reaction products were separated by electrophoresis of 10 μL of PCR products in 1.5% agarose gel in 1× TAE buffer, visualized by staining with ethidium bromide, and examined using an ultraviolet transilluminator. The molecular weight of the obtained product was determined based on the molecular weight marker (Gene Ruler TM 100 bp DNA Ladder [Fermentas, Canada]) [14]. A sample was considered positive when an amplicon of 146 bp was demonstrated.

### RT-qPCR

RT-qPCR was performed in a final volume of 20 μL. The reaction mixture contained 5 μL of DNA, 10 μL of 2× Platinum™ SuperFi™ PCR Master Mix (Invitrogen, Carlsbad, CA), 2.0 μL of Is1Pri_f (10 pmol), 2.0 μL of primer Is1Pri_r (10 pmol), 0.3 μL of probe Tqpro_IS1, and water sufficient to make up the reaction volume. The PCR thermal profile used for optimized conventional PCR was applied, and reactions were carried out using a 7500 Fast Real-Time PCR system (Applied Biosystems, USA) [31].

## Results

**Figure-1:**
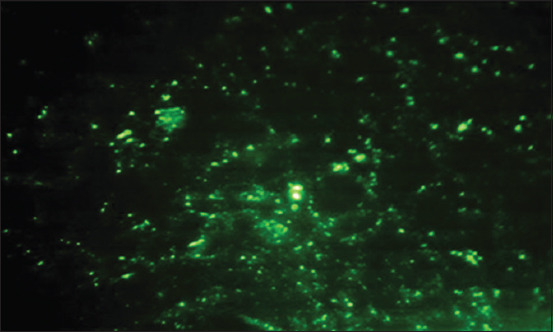
Seroprevalence of *C. burnetii* (IgG antibodies) in human samples as tested by indirect immunofluorescent antibody test (IFAT) IFA.

On the basis of the IFA, the seroprevalence rates of *C. burnetii* infection (IgG and IgM) in cattle, sheep, goats, and humans were 45.3%, 56.0%, 45.7%, and 53.3%, respectively (Table-1 and Figure-1).

**Table-1:** Prevalence of anti-*C. burnetii* antibodies by IFA for the detection of specific IgM and IgG antibodies.

Species	No. of serum samples	No. of positive IFAT%	No. of positive IFAT	

			IgM %	IgG %
Cattle	75	34 45.3%	10 29.4%	2470.6%
Sheep	50	28 56%	8 28.6%	20 71.4%
Goats	35	16 45.7%	6 37.5%	10 62.5%
Human	120	64 53.3%	18 28.1%	46 71.9%
Total	280	142 50.7%	42 29.6%	100 70.4%

IFA=Immunofluorescence assay. *C. burnetii=Coxiella burnetii*

On the basis of the ELISA, the seroprevalence rates of *C. burnetii* infection in cattle, sheep, goats, and humans were 50.7%, 60.0%, 51.4%, and 55.0%, respectively. In humans, the seroprevalence was 54.3% in patients with fever of unknown origin, 30.4% in flu patients, 37.5% in kidney dialysis patients, 77.8% in hepatitis C virus patients, and 62.5% in hepatitis B virus patients (Tables-2-4).

**Table-2:** Prevalence of anti-*C. burnetii* antibodies by ELISA.

Species	No. of serum samples	Positive samples by ELISA test	

		No.	%
Bovine	75	38	50.7
Sheep	50	30	60
Goats	35	18	51.4
Human	120	66	55
Total	280	152	54.3

ELISA=Enzyme-linked immunosorbent assay,*C. burnetii=Coxiella burnetii*

**Table-3:** The prevalence of anti-*C. burnetii* antibodies by IFA for the detection of IgM and IgG antibodies in patients in hospitals in the Assiut district.

Case report	No. of samples	No. of positive IFAT %	H1		H2		H3	
		
			IgM	IgG	IgM	IgG	IgM	IgG
Fever	46	24 52.1%	3/16 18.75%	5/16 31.3%	2/20 10%	8/20 40%	2/10 20%	4/10 40%
Flu	23	7 30.4%	1/5 20%	1/5 20%	2/14 14.3%	2/14 14.3%	1/4 25%	0/4 0%
Kidney dialysis	8	3 37.5%	0/3 0%	1/3 33.3%	1/3 33.3%	1/3 33.3%	0/2 0%	0/2 0%
IHCV	27	20 74.1%	2/8 25%	4/8 50%	3/12 25%	7/12 58.3%	1/7 14.3%	3/7 42.9%
IHBV	16	10 62.5%	1/6 16.7%	2/6 33.3%	2/7 28.6%	3/7 42.9%	1/3 33.3%	1/3 33.3%
Total	120	64 53.3%	7/38 18.4%	13/38 34.2%	10/56 17.9%	21/56 37.5%	5/26 19.2%	8/26 30.8%

IHCV =Infectious hepatitis C virus, IHBV=Infectious hepatitis B virus, H1= Hospital 1, H2= Hospital 2, H3=hospital 3. IFA=Immunofluorescence assay, *C. burnetii=Coxiella burnetii*

**Table-4:** The prevalence of anti-*C. burnetii* antibodies by ELISA in patients in hospitals in the Assiut district.

Case report	No. of samples	No. of +ve ELISA	H1	H2	H3
Fever	46	25 54.3%	9/16 56.3%	10/20 50%	6/10 60%
Flu	23	7 30.4%	2/5 40%	4/14 28.6%	1/4 25%
Kidney dialysis	8	3 37.5%	1/3 33.3%	2/3 66.7%	0/2 0%
IHCV	27	21 77.8%	6/8 75%	10/12 83.3%	5/7 71.4%
IHBV	16	10 62.5%	3/6 50%	5/7 71.4%	2/3 66.7%
Total	120	66 55%	21/38 55.3%	31/56 55.4%	14/26 53.8%

IHCV =Infectious hepatitis C virus, IHBV=Infectious hepatitis B virus, H1= Hospital 1, H2= Hospital 2, H3=Hospital 3. ELISA=Enzyme-linked immunosorbent assay, *C. burnetii=Coxiella burnetii*

The samples positive for anti-*C. Burnetii* antibodies were tested by RT-qPCR in cattle, sheep, and goats (Table-5). Detection of *C. burnetii*-specific DNA in genomic DNA from human blood samples in Assiut Hospitals was also tested by RT-qPCR (Table-6).

**Table-5:** RT-qPCR analysis for *C. burnetii*-specific DNA in seropositive samples. The seropositive samples were subjected to RT-PCR analysis and the ratios were calculated from positive samples of ELISA.

Species	No of +ve samples	Positive samples by PCR	

		No.	%
Cattle	38	12	31.6
Sheep	30	14	46.7
Goats	18	8	44.4
Human	66	45	68.2
Total	152	79	51.9

ELISA=Enzyme-linked immunosorbent assay, RT-qPCR=Real-time quantitative polymerase chain reaction, *C. burnetii=Coxiella burnetii*

**Table-6:** Detection of *C. burnetii*-specific DNA in blood samples from patients admitted to hospitals in the Assiut Governorate. The percentage of *C. burnetii* in hospitals (H1, H2, and H3) and the percentage of *C. burnetii* by RT-PCR were calculated from seropositive samples of ELISA.

Case report	No. of +ve samples	No. of +ve PCR	H1	H2	H3
Fever	25 54.3%	20 80%	7 35%	8	5
Flu	7 30.4%	4 57%	1 25%	2 50%	1 25%
Kidney dialysis	3 37.5%	1 33.3	1 100%	0	0
IHCV	21 77.8%	14 66.7%	4 28.6%	5 35.7%	5 35.7%
IHBV	10 62.5%	6 60%	2 33.3%	3 50%	1 16.7%
Total	66 55%	45 68.2%	15 33.3%	18 40%	12 26.7

ELISA=Enzyme-linked immunosorbent assay, RT-qPCR=Real-time quantitative polymerase chain reaction, *C. burnetii=Coxiella burnetii*

**Figure-2:**
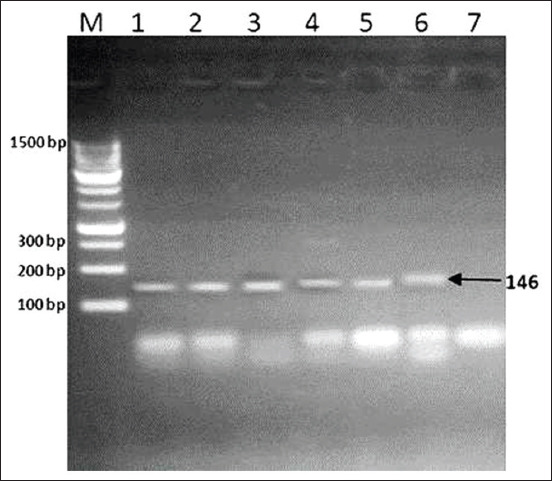
Agarose gel electrophoresis of polymerase chain reaction amplicons (146 bp) in samples positive for anti-*Coxiella burnetii* phase II IgG antibodies. Lane M: 100 bp molecular size DNA marker. Lanes 1 and 2: Positive sheep whole-blood samples, lanes 3 and 4: Positive goat whole-blood samples, lanes 5 and 6: Positive human whole-blood samples, and lane 7: Negative control.

Figure-2 presented the Agarose gel electrophoresis of PCR amplicons (146 bp) in samples positive for anti-C. burnetii phase II IgG antibodies. Lane M: 100 bp molecular size DNA marker. Lanes 1 and 2: Positive sheep whole-blood samples, lanes 3 and 4: Positive goat whole-blood samples, lanes 5 and 6: Positive human whole-blood samples, and lane 7: Negative control.

## Discussion

Q fever is a zoonotic disease caused by the bacterium *Coxiella burnetii*. A Q fever outbreak was documented in Australia in 1935, and subsequently, the infection has been spreading [32,33]. Q fever in humans may present as a flu-like illness with symptoms such as headache, myalgia, and/or atypical pneumonia. Symptoms such as hepatitis and endocarditis may be long lasting in chronic cases [20]. The prevalence of Q fever is higher in females than in males. In addition, a higher seropositivity rate has been found in pregnant farm animals [34].

*C. burnetii*-specific antibodies have been detected in most livestock species of significance to Egypt. Therefore, current and future research should focus particularly on camels and buffalos in order to better map the transmission mechanism of *C. burnetii* to humans and identify any possible risk factors linked to exposure [20].

The prevalence of *C. burnetii* infection in animals varies widely according to the species tested, geographic location, and diagnostic test used [35]. In the present study, the ELISA test revealed seropositive rates of 50.7% in cattle, 60.0% in sheep, 51.4% in goats, and 55% in humans (Table-2).

IFA is one of the most commonly used serological approaches to diagnose *C. burnetii* infection [36]. ELISA has been proven to have a sensitivity similar to that of IFA [36], which was also documented in the present study. Therefore, both of them can be used for serological diagnosis.

In the current study, anti-*C. burnetii* antibodies were detected by IFA in 45.3% of the cattle, 56.0% of the sheep, 45.7% of the goats, 53.3% of the apparently healthy humans, 52.1% of patients with fever of unknown origin, 30.4% in flu patients, 37.5% in kidney dialysis patients, 74.1% in hepatitis C virus patients, and 62.5% hepatitis B virus patients. The IFA is the most specific and sensitive test for phase II and phase I IgG antibodies, and for phase II and phase I IgM antibodies. The present work revealed that the presence of *C. burnetii* antibodies in hospitalized patients in Assiut Governorate by IFAT to be (53.3%). Most of those patients had contact with animals and came from rural areas. Clinical manifestations included high temperature, chills, and bradycardia. Seropositivity may involve testing positive for antibodies from phase II and phase I *C. burnetii* infection. Phase II antibodies are more prevalent during acute infection, and chronic infection is characterized mainly by a phase I antibody response [36]. Results of the present work agree with the studies conducted in Egypt [37] and Sudan [38].

Infected small ruminants may shed high numbers of bacteria in their excretions, such as milk and feces. Animals may be shedding bacteria and/or remain seropositive long after the acute infection [39,40]. Other authors reported significantly higher seroprevalence in goats than in cattle [41].

The results of the ELISA tests indicated the high prevalence of *C. burnetii* infection in people in Ilam Province. Further, they demonstrate high seroprevalence of endemic Q fever in the countryside and in Bedouin populations. Furthermore, higher prevalence has been detected among people who have close engagement with livestock due to their occupations, such as animal husbandry workers (45.13%) [42].

In the present study, 60 out of 150 (40.4%) animals included tested positive for specific antibodies; a proportion that is close to that reported in humans.

There is also an agreement between the results of this study and the results of two similar studies performed in Turkey, where the *C. burnetii* seroprevalence reached 50.9% among people with close contact to livestock. Abattoir workers, farmers, and butchers were found to have the highest prevalence rates (32.8%) [43,44].

Close contact with domestic animals has been reported to be one of the key risk factors related to the transmission of *C. burnetii* to humans [45,46]. Inhaling polluted materials from feces of infected animals or their urine, milk, and birth products is considered a key route of infection [47].

The seroprevalence of *C. burnetii* infection among slaughterhouse workers and butchers was found to be 22.5% in a study performed in 2016 in the Provinces of Sistani and Baluchistan, which are located in the Southeast of Iran [48].

In another study in Kurdistan Province, west of Iran, the seroprevalence of *C. burnetii* was reported 27.83% among hunters and their members of the family, butchers, employees of the public health centers, and patients who referred to laboratories [46]. In addition, high seroprevalence of *C. burnetii* infection was according in studies conducted in neighboring countries of Iran, particularly within the west and northwest of the country [43,44].

Age and close contact with goats, rodents, and cats, as well as mosquito bites, are considered risk factors of *C. burnetii* seropositivity. Livestock, including goats, cattle, and sheep, are considered the most substantial animal reservoirs for human infection and considered the key reservoirs for urban outbreaks of Q fever [49].

This demonstrated the risk of *C. burnetii* infection among the Bedouin and countryside populations, who have extensive exposure to livestock [50].

Further, the results of the study detect and demonstrate high seropositive cases of Q fever in people who raise sheep, cattle, and goats; the Bedouin and countryside populations, and people who are in close contact with livestock due to their occupation. The existence of infected ticks in different parts of Iran has been demonstrated and reported in a couple of studies [51,52]. For example, *C. burnetii* was detected in 160 ticks gathered from domestic livestock (including goats and sheep) in 2009 in Kerman Province in the Southeast of Iran [53].

In a study performed on Namibian blood donors in 2014, a two-fold higher risk of infection was identified for those exposed to domestic animals recently diagnosed with tick-borne fever (40%) than populations exposed to domestic animals negative for tick-borne fever (22.9%) [54].

Q fever outbreaks have been detected in various regions of Europe in both animals and people. Moreover, people exposed to animals through their profession have a higher risk of infection with *C. burnetii* than others. For example, high prevalence rates of *C. burnetii* infections among farmers tested using serological tests such as ELISA and IFA have been reported (31.12% and 39.07%, respectively) [24].

Age, place [55] (e.g., the countryside, where farmers are most often exposed to animals), and illiteracy (or low education) [56] are among the key factors contributing to a higher risk of *C. burnetii* infection in humans. For example, in Switzerland, individuals older than 15 years were reported to be at a higher risk (five-fold increased risk) than the younger respondents [55]. It was also reported that low education level is among the key risk factors contributing to an increased risk of developing Q fever [56].

Therefore, it could be assumed that raising the level of awareness regarding *C. burnetii* infection and its mode(s) of transmission could contribute to minimizing the prevalence of the infection.

Among the limitations of this study was its inability to infer valid conclusions regarding associations between risk factors and health outcomes.

The ELISA test detected anti-*C. burnetii* antibodies in 54.3% of patients with fever of unknown origin, in 77.8% of patients with hepatitis C virus, and in 62.5% of patients with hepatitis B virus. IFA and ELISA yielded similar seroprevalence data in patients undergoing kidney dialysis and patients with flu-like disease (37.5% and 30.4%, respectively) as they are seropositive *C. burnetii*. The IFA detected antibodies in 52.1% of the patients with fever of unknown origin, 77.8% of those with hepatitis C virus, and 62.5% of patients with hepatitis B virus. Hence, the seroprevalence in patients with chronic hepatitis C and hepatitis B virus infection with *C. burnetii* was remarkably high. Both variations of the hepatitis virus and *C. burnetii* cause liver cirrhosis.

This result was supported by a study [57] reporting that acute Q fever can be considered a possible cause of acute renal failure. In addition, other authors identified chronic Q fever in two dialysis patients [58].

The seroprevalence data pertaining to the patients with fever of unknown origin are supported by a recent study [59], which reported the case of a 53-year-old man diagnosed with fever of unknown origin who had been subjected to F-18 fluorodeoxyglucose positron emission tomography/computed tomography.

With regard to hepatitis, the results of the present study are interesting in the context of a recent study [60], where it was reported that 16 (27.6%) out of 58 patients diagnosed with acute Q fever hepatitis were found to have viral hepatitis (hepatitis B virus infection in 12 and hepatitis C virus infection in 4). Patients with Q fever have been found to have abnormal liver function. This is an issue of great concern for regions that are endemic to Q fever hepatitis as well as viral hepatitis [61]. A study stated that one out of nine patients with Q fever was diagnosed with jaundice, which suggests the involvement of the liver in Q fever [62].

In 1956, the finding of a granuloma-like lesion was first described in the above-mentioned study [61] using a needle biopsy on patients diagnosed with Q fever and liver involvement.

After that study, various other studies have also described the pathological features of Q fever hepatitis, referred to as doughnut lesions, which exist in the form of inflammatory tumor changes with fibrinoid rings as well as clear central spaces [60,63,64].

The presence of Q fever in 17 (13.3%) out of 128 patients diagnosed with infectious hepatitis was reported in the study by Alkan *et al*. [65]. The study further revealed that acute headache appeared to be the sole symptom, which could be used to discriminate Q fever from infectious hepatitis, but this discerning trait was not particularly specific. Apart from the problem of differentiating between Q fever, noninfectious hepatitis, and infectious hepatitis, there is still an urgent need to explore whether there are explicit differences in clinical manifestations, biochemical test values, and responses to treatment among patients who have underlying viral hepatitis. This is an issue of crucial importance, especially in countries where viral hepatitis is endemic, including Egypt. Similarly, differentiation between Q fever, noninfectious hepatitis, and infectious hepatitis is also of crucial importance.

Different studies show different rates of infection of *C. burnetii* infection in different countries. The seropositivity of *C. burnetii* infection in Japanese bovine herds reached 84.3% and was suggested to lead to reproductive disorders [18]. Other prevalence rates reached 14.39% in sheep in China and 13.5% in sheep in Northeastern China [1]. In Chad, the *C. burnetii* seropositivity values were 16% in humans, 13% in cattle, 23% in goats, and 33% in sheep [66]. The prevalence of *C. burnetii* infection in humans occupationally exposed to animals in Poland was 31.12% [67].

Q fever may lead to reproductive disorders as exemplified by a study emphasizing the existence of Q fever in all of the three major significant domestic ruminant species in Bangladesh [68]. It might play a key role in abortions in sheep because the seroprevalence is relatively high [7]. In the current study, quite a few serum samples from animals and humans showed anti-*C. burnetii* antibody positivity. IFA results were further confirmed by RT-qPCR. We evaluated the performance of RT-qPCR for the detection of *C. burnetii-*specific DNA targeting the IS1111 gene using serum samples from patients with acute Q fever. The use of molecular detection based on the IS1111 gene has 100% specificity [69]. Not all seropositive samples were positive by RT-PCR. This agrees with a recent study in which it was stated that serologic techniques may be hampered by limited accuracy because some animals may pose a risk for infection before the development of antibodies by shedding the bacteria and a few of the infected animals might never seroconvert [70].

Confirmatory testing by RT-qPCR revealed a proportion of PCR-positive samples among those that had tested positive by ELISA of 51.9% (cattle, 31.6%; sheep, 46.7%; goats, 44.4%; and humans, 68.2%). The calculated proportions from the total numbers of samples were 16% in cattle, (31.6%) in sheep, (28%), (26.7%) in goats, and (37.5%) in humans.

*C. burnetii* real-time qPCR positivity was used on both acute-phase and follow-up convalescent-phase serum samples out of 65 serum samples with acute Q fever. Classification of sera was performed based on antibody profiles according to the IFA. We observed that PCR was positive in 49/50 (98%) seronegative sera, 9/10 (90%) sera with isolated IgM-II antibodies, and 3/13 (23%) sera with IgM-II antibodies [71]. Therefore, RT-PCR should be used to confirm seronegative samples in future studies to improve the accuracy in diagnosing Q fever.

A study provided data on PCR-based detection of *C. burnetii* in blood samples from small ruminants. ELISA and PCR were used to detect *C. burnetii* in native Korean goats (*Capra hircus coreanae*) for the first time. Out of 597 goats, 114 (19.1%; 95% confidence interval [CI] = 16.1-22.4) and 57 (9.5%; 95% CI = 7.5-12.2) were *C. burnetii* positive by ELISA and PCR, respectively [72].

A study was undertaken in Northwest of Iran (or West of Azerbaijan). The main aim of that study was to identify the prevalence of *C. burnetii* in 840 raw milk samples gathered from domestic animals such as buffalos and cattle. DNA was extracted from all milk samples, and nested PCR was employed to detect *C. burnetii* using IS1111 as the target. It was shown that 16.9% of the milk samples (19.3% of the buffalo and 14.6% of the cattle samples) tested positive for *C. burnetii*, which is in agreement with the results of our study on cattle (16%) [73].

*C. burnetii* using RT-qPCR as well as serological surveys of animals is important methods for the diagnosis and control of Q-fever [27,74].

RT-PCR rapidly screen samples in local outbreaks for other organisms relevant for humans or animals sensitivity of real-time PCR was high after testing samples from a local Q-fever outbreak [68].

Finally, in Egypt, employing immunofluorescence assay (IFA) or ELISA for the diagnosis of *C. burnetii* and confirming it by (RT-qPCR) are important methods for the diagnosis and control of Q-fever. RT-PCR should be used to confirm seronegative samples in future studies to reach accurate early diagnosis of the Q fever.

Future research could include wide-scale surveys of patients to identify relationships between Q fever, hepatitis B, hepatitis C and liver cirrhosis, fever of unknown origin, and reproductive disorders, such as habitual abortion, which may be linked to *C. burnetii* infection.

## Conclusion

Apparently healthy cattle, sheep, and goats may be important reservoirs of *C. burnetii* infection. Although hepatitis is one of the major manifestations of acute Q fever, the influence of viral hepatitis on Q fever remains to be properly investigated. Q fever is not a reportable disease in Egypt, and clinical cases are probably unrecognized by the health-care system. More information on the epidemiology of *C. burnetii* in Egypt is warranted, as well as related topics such as distribution, pathogenesis, and molecular typing. IFA and confirming it by (RT-qPCR) are important methods for diagnosis and control of Q-fever in animals and human.

## Authors’ Contributions

HA, performed the practical part, contributed to the analysis and interpretation of data and writing the original draft. MMS contributed in the part of PCR and in reviewing the paper. SAKS contributed in reviewing the paper. MAE contributed in the collection of samples of hepatitis and reviewing the paper. ME contributed in reviewing the paper. HHA contributed in writing and organization of the whole paper in final form, reviewing and English editing of the paper. All authors have read and approved the final manuscript.

## Data Availability

All data collected during this study are included in this article.
